# Cardiac Tissue Engineering for the Treatment of Myocardial Infarction

**DOI:** 10.3390/jcdd8110153

**Published:** 2021-11-08

**Authors:** Dongmin Yu, Xiaowei Wang, Lei Ye

**Affiliations:** 1 Department of Cardiovascular Surgery, The First Affiliated Hospital with Nanjing Medical University, Nanjing 210029, China; yudongmin@stu.njmu.edu.cn; 2 National Heart Research Institute Singapore, National Heart Centre Singapore, 5 Hospital Drive, Singapore 169609, Singapore

**Keywords:** engineered cardiac tissue, myocardial infarction, stem cells

## Abstract

Poor cell engraftment rate is one of the primary factors limiting the effectiveness of cell transfer therapy for cardiac repair. Recent studies have shown that the combination of cell-based therapy and tissue engineering technology can improve stem cell engraftment and promote the therapeutic effects of the treatment for myocardial infarction. This mini-review summarizes the recent progress in cardiac tissue engineering of cardiovascular cells from differentiated human pluripotent stem cells (PSCs), highlights their therapeutic applications for the treatment of myocardial infarction, and discusses the present challenges of cardiac tissue engineering and possible future directions from a clinical perspective.

## 1. Introduction

Ischemic heart disease (IHD) is a leading cause of death worldwide and a major contributor to the global health and economic burden. In 2017, there were a total of 126.5 million reported cases of IHD, 10.6 million newly diagnosed cases, and 8.9 million deaths worldwide [1]. Although the current diagnosis and treatment of cardiovascular diseases (CVDs) are well developed, IHD is still associated with high morbidity and mortality rates, suggesting an urgent need to develop novel preventive and treatment strategies for IHD. 

Ischemic heart disease causes myocardial infarction (MI), which results in the death and loss of cardiomyocytes (CMs). Apoptosis, necrosis, and autophagy in CMs are the typical hallmarks of cardiac pathology in MI [2]. The loss of morpho-functionally competent CMs in the infarcted myocardium results in a vicious cycle of left ventricular (LV) remodeling and heart failure (HF) [3]. MI-induced HF has a significant impact on mortality [4]. In fact, for patients with a history of MI, HF increases total mortality risk by three-fold and cardiovascular mortality risk by four-fold.

The extent of cardiac tissue healing and regeneration after ischemic injury is limited. Cell-based transfer therapy is currently being tested and has shown promising results for reducing myocardial damage and the restriction of heart remodeling after MI [5]. However, clinical studies have failed to show significant improvement thus far [6]. A growing body of evidence suggests that the therapeutic benefits of cell implantation are mainly due to the paracrine factors released by implanted cells instead of the engrafted cells, as only a small portion of these cells survive in the infarcted myocardium [7]. Recent studies have shown that the combination of cell-based therapy and tissue engineering technology can improve stem cell engraftment, leading to increased therapeutic effects [8,9]. Engineered cardiac tissue from the derivatives of human induced pluripotent stem cells (hiPSC) can not only develop functional cardiac tissue for cell transfer therapy, but also can be used for modeling heart disease and screening drugs in vitro [10,11].

This mini-review will summarize the recent progress in tissue engineering of cardiovascular cells from differentiated hPSCs and their therapeutic applications for the treatment of MI. Finally, the review will also discuss the current challenges of cardiac tissue engineering and possible future directions from a clinical perspective.

## 2. Types of Progenitor or Stem Cells Used for the Treatment of Myocardial Infarction

Cardiac tissue engineering aims to replace fibrotic scars by creating contractile and functional heart tissues. A wide variety of stem cells, their derivatives, and progenitor cells are currently being tested for these purposes. Stem cells can be divided into two types according to their sources: embryonic stem cells (ESCs) and adult progenitor/stem cells [12]. In terms of their differentiation capabilities, stem cells can be divided into four main types: totipotent stem cells, PSCs, multipotent stem cells, and unipotent stem cells [12]. The major progenitor/stem cells used in the field of cellular cardiomyoplasty are myoblasts [13,14,15], mesenchymal stem cells [16,17,18], ESCs [19,20], and iPSCs [21,22,23]. The advantages and disadvantages of each cell type are summarized in Table 1. Since ESCs and iPSCs can be differentiated into cardiovascular cells, including cardiac progenitor cells (CPCs), CMs, endothelial cells (ECs), and smooth muscle cells (SMCs), these stem cells may be used for cardiac tissue engineering and are being extensively investigated. 

Mouse embryonic stem cells (mESCs) were established by two research teams led by Martin Evans and Matthew Kaufman in 1981 [12]. On the other hand, human ESCs (hESCs) were established by James Thomson in 1998 [12]. Mouse iPSCs (miPSCs) were established by Yamanak in 2006 using octamer binding transcription factor 3/4, sex determining region y-box 2, the cellular-myelocytomatosis viral oncogene, and kruppel-like factor 4 transcription factors from mouse fibroblasts [12]. The following year, Yamanak and James Thomson independently and simultaneously reported the establishment of hiPSCs from human dermal fibroblasts [12]. Generally, miPSCs or hiPSCs are almost identical to their counterpart, mESCs or hESCs. 

PSCs are classified into two distinct states: naïve and primed pluripotent states. Naïve PSCs include mESCs and miPSCs, while primed PSCs include mouse epiblast stem cells (mEpiSCs), hESCs, and hiPSCs [24]. Although both naïve and primed PSCs are capable of self-renewal and can differentiate into the three germ layers in vitro and in vivo, only naïve PSCs can generate germline-competent chimeras in vivo [25].

Recent studies demonstrate that hiPSCs are a powerful tool for modeling diseases and treating various human diseases, including cardiovascular diseases, degenerative neuronal diseases, and diabetes, due to their unique advantage (Table 1). Like hESCs, hiPSCs have self-renewal capabilities and differentiate into all somatic cells found in the body regardless of their tissue of origin. hiSPCs are therefore being extensively explored to develop functional tissues for the treatment of myocardial injury. 

In the following section, we will discuss the ongoing progress of tissue engineering in PSC reprogramming and differentiation and highlight the application of tissue engineering using cardiovascular cells derived from hPSCs to treat MI in animal models. 

## 3. Application of Tissue Engineering in PSC Reprogramming, Differentiation, and Treatment of Myocardial Infarction

Biomaterial scaffolds, which mimic the natural extracellular matrix (ECM) environment, have been extensively investigated for the engineering of cardiac tissues for PSC reprogramming, differentiation, and treatment of MI.

### 3.1. Tissue Engineering for the Regulation of PSC Reprogramming

Some biomaterials have been shown to improve reprogramming efficiency by changing epigenetic barriers. Downing et al. showed that the microgroove substrates increased the reprogramming efficiency of fibroblasts into iPSCs [26]. Notably, micro-grooved surfaces increased histone H3 acetylation and methylation, overcoming epigenetic barriers in reprogramming [26]. A study using tumor-initiating stem-like cells showed that a matrix made of fibrin gel promoted H3K9 demethylation and Sox2 expression [27], both of which are known to be involved in iPSC reprogramming [28,29]. 

An engineering technique, such as the clustered regularly interspaced short palindromic repeats (CRISPR)/Cas9 system is sought as a preferred method for the reprogramming and genetic modification of PSCs [30,31]. CRISPR/Cas9-based gene activator (CRISPRa), which has a high multiplexing capacity and direct targeting of endogenous loci, has also been used for cellular reprogramming [32]. Weltner et al. showed that using CRISPRa reduces the reprogramming efficiency of human dermal fibroblasts [32]. Moreover, Howden et al. developed a protocol that combined the CRISPR/Cas9 system and the enhanced episomal-based reprogramming to simultaneously generate gene-edited and passage-matched unmodified iPSC lines through the electroporation of human fibroblasts [33]. Compared to human fibroblasts, erythroblasts are later found to be a better cell candidate for simultaneous reprogramming and gene-editing using CRISPR/Cas9 [34]. These studies suggest that CRISPR/Cas9 technique may be a better option for iPSC reprogramming and gene editing. 

### 3.2. Tissue Engineering for the Differentiation of PSCs into Cardiovascular Cells

Recent advances in tissue engineering allow for the designing of cellular structures or the incorporation of molecules to control the mechanical force and the release of certain factors that affect the differentiation of PSCs. Poh et al. developed a method to generate germ layers from a single line of mESCs [35]. In this study, an embryoid colony formed from a single mESC in 3D matrix, which was then cultured in 1 kPa collagen-1-coated 2D substrates. This resulted in the self-organization of the ectoderm, mesoderm, and endoderm layers in vitro.

We showed that a 3D environment promoted the differentiation of hiPSC into ECs when hiPSCs were seeded into fibrinogen/thrombin scaffolds [36]. A 3D environment upregulated the expression of p38 mitogen-activated protein kinase and extracellular signal-regulated kinase 1/2 signaling pathways [37]. Thus, the use of U-46619, a prostaglandin H2 analog that activates ERK1/2 and p38 MAPK signaling pathways, effectively increased EC differentiation efficiency (>85%) [37].

When hiPSC-ECs were cultured in peptide-functionalized poly(ethylene glycol) (PEG) hydrogels, polygonal vascular networks were formed [38]. Vascular networks with lumens were stable for at least 14 days. A microcarrier (MC) suspension agitated platform has been shown to differentiate hESCs and hiPSCs into CMs [39]. Agitation results in homogenous and hydrodynamic shear stress, therefore inducing CM differentiation. The resulting yield is 38.3% and 39.3% higher for Troponin T and myosin heavy chain, respectively, than static culture control. Electrospun polylactide-glycolic acid/collagen (PLGA/Col) scaffolds have been used to differentiate ESCs to CMs [40]. Better interaction and growth of differentiated ESCs were observed on the PLGA/Col scaffolds relative to PLGA-only scaffolds. On the other hand, a cryopreserved amniotic membrane has been shown to direct the differentiation of hiPSC-derived cardiac progenitor cells to CMs in the presence of cytokines [41]. Amniotic membranes increased the expression of cardiac transcription factors and myofibril proteins, accelerated the intracellular calcium transients, and enhanced the mitochondrial complexity formation in CMs. These studies suggest that some biomaterials can be designed to provide PSCs with essential mechanical and chemical cues to direct their differentiation.

### 3.3. Tissue Engineering Using PSC-Derived Cardiovascular Cells for the Treatment of MI

It has been established that poor cell engraftment rate is one of the primary factors limiting the effectiveness of cell transfer therapy for cardiac repair. The low engraftment rate can be improved by combining the technique with cardiac tissue engineering, in which cells are transplanted within a supporting matrix [8]. Biomaterials can provide mechanical support for the stem cells and supply nutrients and oxygen to encapsulated cells [9,10,41,42]. 

Cardiac tissue engineering combines cells and biological scaffolds to make heart grafts to replace the damaged heart tissue and restore or improve the heart structure and function. In addition to cellular cardiomyoplasty, heart valve reconstruction, and vessel graft manufacturing are also the main target areas of heart tissue engineering. 

Common tissue engineering materials are made of either natural or synthetic biomaterials. Natural biomaterials include Matrigel [42,43,44], hyaluronic acid [45,46], gelatin [47,48,49], chitosan [50,51], alginate [52,53,54], collagen [42,55,56,57,58,59], fibrin [21,22,23,55,60,61,62,63], elastin [64], amniotic membrane [41], and spider silk proteins [65]. Synthetic biomaterials, on the other hand, include poly(ethylene glycol) (PEG) [66,67], polylactide-glycolic acid (PLGA) [68,69], polyacrylamide (PAA) [70], poly(N-isopropylacrylamide) (PNIPAAm) [71], polycaprolactone (PCL) [72], and polyurethane (PU) [73]. More recently, natural/synthetic hybrid hydrogels are being developed as an alternative biomaterial that combines natural and synthetic materials through covalent grafting or crosslinking [74,75].

#### 3.3.1. Natural Biomaterials

Hydrogels made of natural biomaterials have high flexibility and water content and provide mechanical support for cell attachment, migration, differentiation, and even proliferation. In cardiac tissue engineering, hydrogels are usually used to inoculate encapsulated cells and compress them into 3D tissues. Natural biomaterials are similar to ECM, and their physiological properties make them suitable for an array of applications.

Although early studies showed the feasibility of direct hydrogel injection for MI treatment [76,77], there is still a need for a biomaterial capable of supporting co-cultured cardiovascular cells for tissue engineering since the cardiac tissue is composed of a variety of cells. Collagen and fibrin are two of the most often used natural biomaterials for manufacturing engineered cardiac tissues (ECTs). This is usually done by culturing PSC-derived cardiovascular cells in biomaterials to treat MI in small (Table 2) and large animal models (Table 3).

Collagen has been used to manufacture ECTs using hESC- or hiPSC-derived cells [42,55,56,57,58,59]. Nakane et al. characterized the impact of cell composition on hiPSC-derived engineered cardiac tissue [59]. In this study, ECTs (15 × 15 mm) made of collagen and hiPSC-CMs, hiPSC-ECS, and hiPSC derived vascular mural cells(total cell number = 6 × 10^6^) displayed the lowest number of dead cells, as well as the lowest degree of maturation. Furthermore, myofiber bundles (d = 0.5 mm) with better cell alignment and higher active stress were also obtained in the same set of cells. In contrast, a higher cell number (>6 × 10^6^) increased cell death and lowered active stress. Moreover, ECTs (developed using collagen/Matrigel hydrogel seeded with hESC-CMs or hiPSC-CMs and fibroblasts) implanted into infarcted rat hearts effectively recovered the myocardial structure and function with reduced scar size [43]. This engineered tissue displayed structures and functions close to the human postnatal myocardium and is therefore functional for cardiac repair. 

Fibrin is another natural biomaterial that has been extensively used to manufacture cardiac tissue [21,22,23,55,60,61,62,63,78]. hiPSC-ECs or hiPSC-CMs have been co-cultured in fibrin gel to create cardiac patches, which were then implanted into infarcted rat hearts [60,61]. Although revascularization was improved in both studies, only the cardiac patch made of hiPSC-CMs and pericytes significantly increased left ventricular (LV) systolic function after MI. These suggest that although rebuilding the vessel and perfusion network in infarcted myocardium is important, these are still insufficient to achieve contractility improvement in injured hearts and that myocytes are still needed to repopulate the fibrotic tissue. Thus, cardiac patches combining fibrin gel with hiPSC-CM and hiPSC-ECs and/or hiPSC-SMCs have been manufactured to address these challenges [63,79,80,81]. Human ECT strips made of fibrin patch loaded with hiPSC-CMs and hiPSC-ECs have been used to treat cryo-injury in guinea pig hearts [63,79]. Some grafts showed vascularization and electrical coupling to the intact heart tissue with improved LV function [79]. Furthermore, electrocardiograms recorded over a 28-day follow-up showed that the rate of arrhythmic events did not differ between the ECT implant and cell-free scaffold implant and the control groups [63].

In another study, the loading of insulin-like growth factor 1 (IGF1) on cell-free fibrin scaffolds on infarcted myocardium has also been shown to increase the engraftment rate (up to 9%) of hiPSC-CM, hiPSC-EC, and hiPSC-SMC in a porcupine I/R model [21]. The seeding of these cells into a fibrin gel and its implantation into a pig heart I/R model has also significantly improved LVEF and reduced apoptosis, myocardial wall stress, infarct size, and hypertrophy in the board-zone myocardium [81]. In addition to this, the efficacy of angiomyogenesis using hiPSC-CMs for transient overexpression of angiopoietin-1 (Ang-1-hiPSC-CMs) has also been determined through the use of fibrin gels. In this study, Ang-1-hiPSC-CMs were seeded into a fibrin/thrombin patch and applied onto the infarcted rat myocardium [22]. Patch transplantation effectively induced host (rat) and donor (human) CM mitosis and arteriole formation, improved the cell engraftment rate, limited the LV dilation, and improved the LV systolic function.

**Table 2:** Tissue engineering using PSC derived cardiovascular cells for treatment of MI in small animal models.

Stem Cell Types	Model	Animal	Biomaterials	ROA	Reference
hESC-CMs	I/R	Rat	Matrigel + collagen	Applied on epicardium	[42]
hESC-CM or hIPSC-CM + fibroblasts	MI	Rat	Matrigel + collagen	Applied on epicardium	[43]
hESC-CMs	MI	Rat	Collagen	Applied on epicardium	[57]
hESC-CM, hiPSC-CM, HUVEC, MSC, and MEF	Uninjured heart	Rat	Collagen	Applied on epicardium	[58]
hiPSC-CM, hiPSC-ECs, and hiPSC-SMCs	MI	Rat	Collagen	Applied on epicardium	[59]
hESC-SSEA-1 + progenitor cells	MI	Rat	Fibrin/thrombin	Applied on epicardium	[82]
hiPSC-CMs	MI	Mouse	Cells, CHIR99021, and FGF1 loaded into fibrin/thrombin scaffold	Applied on epicardium	[83]
hiPSC-ECs and pericyte	MI	Rat	Fibrin/thrombin	Applied on epicardium	[60]
hiPSC-CMs and pericytes	MI	Rat	Fibrin/thrombin	Applied on epicardium	[61]
Ang-1 modified hiPSC-CMs	MI	Rat	Fibrin/thrombin	Applied on epicardium	[22]
hhiPSC-CMs	Cryo-injury	Guinea pig and pig	Fibrin/thrombin	Applied on epicardium	[62]
hiPSC-CMs and hiPSC-ECs	Cryo-injury	Guinea pig	Fibrin/thrombin	Applied on epicardium	[79]
hiPSC-CMs and hiPSC-ECs	Cryo-injury	Guinea pig	Fibrin/thrombin	Applied on epicardium	[63]
hiPSC-CMs, hiPSC-ECs, and hiPSC-SMCs	MI	Mouse	Gelatin	Applied on epicardium	[47]

FGF1, fibroblast growth factor-1; hEShESC-CM, human embryonic stem hiPSC-CM, human induced pluripotent stem cells derived cardiomyocytes; hiPSC-ECs, human induced pluripotent stem cells derived endothelial cells; hiPSC-SMCs, human induced pluripotent stem cells derived smooth muscle cells; HUVEC, human umbilical vein endothelial cells; I/R, ischemia/reperfusion; MEF, mouse embryonic fibroblasts; MI, myocardial infarction; MSC, mesenchymal stem cells; ROA, route of administration; SSEA-1, stage-specific embryonic antigen-1.

**Table 3:** Tissue engineering using PSC derived cardiovascular cells for treatment of MI in large animal models and clinic.

Stem Cell Types	Model	Animal	Biomaterials	ROA	Reference
hiPSC-CM	Chronic ischemia	Pigs	hiPSC-CM sheet	Applied on epicardium	[84]
hiPSC-CM	Chronic ischemia	Pigs	Omentum flap	Intramyocardial injection of cells + omentum flap applied on epicardium	[85]
hiPSC-CM and hMSC	Chronic ischemia	Pigs	hiPSC-CM and hMSC sheet; Omentum flap	Applied on epicardium	[86]
hiPSC-EC, and hiPSC-SMC	I/R	Pigs	Fibrin/thrombin	Applied on epicardium	[87]
hiPSC-CM, hiPSC-EC, and hiPSC-SMC	I/R	Pigs	Fibrin/thrombin	Intramyocardial injection of cells + IGF-1 loaded fibrin/thrombin scaffold applied on epicardium	[21]
hiPSC-CM	MI	Pigs	Gelatin and fibrin/thrombin	hiPSC-CM intramyocardial injection; Gelatin microspheres for sustained release of Tb4; Fibrin/thrombin scaffold applied on epicardium	[23]
hiPSC-CM	MI	Micro mini-pigs	Gelatin	Applied on epicardium	[88]
hESC-SSEA-1^+^	I/R	Patients	Cells were cultured in fibrin/thrombin patch	Applied on epicardium in adjunction to CABG	[89,90]

CABG, coronary ratery bypass graft; hESC-SSEA1, human embryonic stem cells derived stage-specific embryonic antigen-1 progenitor cells; hiPSC-CM, human induced pluripotent shiPSC-ECs, human induced pluripotent stem cells derived endothelial cells; hiPSC-SMCs, human induced pluripotent stem cells derived smooth muscle cells; hMSCs, human mesenchymal stem cells; I/R, ischemia/reperfusion; IGF-, insulin-like growth factor-1; ROA, route of administration.

To meet clinical requirements, good manufacturing practices to improve the quality of human ECT have been developed by Querdel et al. [62]. Specifically, they established protocols for manufacturing clinical-grade mesh-structured tissue patches using collagen and hiPSC-CMs. ECTs with three different doses (4.5 × 10^6^, 8.5 × 10^6^, and 12 × 10^6^) of hiPSC-CMs were tested in a cryoinjury guinea pig heart model [62]. Heart tissue transplantation resulted in a dose-dependent remuscularization; however, only high-dose patches improved LV function with partial remuscularization observed in injured hearts. 

More recently, amniotic membranes and spider silk proteins have also been investigated for ECT manufacturing [41,65]. Amniotic membranes promoted the differentiation of hiPSC-derived cardiac progenitor cells into CMs and enhanced the intracellular calcium transients and the cellular mitochondrial complexity of differentiated CMs [41]. Spider silk films have also been demonstrated to support the growth of hiPSC-CMs [65]. It has been found that films can contract up to 14 days in vitro. Moreover, hiPSC-CMs on films also respond to pharmacological stimulation, including phenylephrine and verapamil. 

#### 3.3.2. Synthetic Materials

The physical and biochemical properties of synthetic materials are easy to control, usually through the modulation of water affinity and degradation rate. PEG is being used as a matrix in many fields of tissue engineering due to its low protein adsorption and inert surface, which then reduces the rate of inflammation after implantation. Its modified form has been used as an injectate for the treatment of MI to limit the adverse post-infarct LV remodeling [66]. PEG hydrogels containing hiPSC-CMs and erythropoietin (EPO) were injected into the infarcted myocardium of a rat MI model [67]. Attenuation of LV remodeling was observed in all groups that received PEG injections. However, LVEF was only significantly increased in the gel-EPO, cell, and gel-cell-EPO groups. PEG was modified with the Arg-Gly-Asp (RGD) peptide to improve cell adhesion, survival, and proliferation, significantly increasing the viability of encapsulated mESC-CMs [91]. The implantation of modified PEG carrying mESC-CMs improved sustained cardiac function (up to 12 weeks) as observed in a mouse MI model. 

Considering the features of myocardial tissue, a biocompatible and biomimetic platform is needed for CM culture and for forming a functional myocardium. Biomimetic cardiac patches made of microgrooved thin PLGA films with hESC-derived CMs have been developed by Chen et al. [68]. This cardiac patch recapitulated the anisotropic electrophysiological feature of native cardiac tissues and is more refractory to premature stimuli than non-grooved PLGA films. Song et al. developed cardiac cell sheets by combining direct cardiac reprogrammed cells with a nanothin membrane made of PLGA [69]. The cell sheets were laid layer-by-layer and prevacularized with endothelial cells between the layers. The implantation of the prevascularized, multilayered cell sheets improved cardiac function and caused a reduction in adverse cardiac remodeling in rat MI models. PNIPAAm, a pPAAm derivative, is a thermosensitive polymer with a thermal transition temperature of 32 °C [92]. Unlike other polymers, it retains its liquid state at room temperature and polymerizes at 37 °C [92]. This unique property has enabled PNIPAAm to be used for direct intramyocardial injection and for manufacturing thermoresponsive cell culture surfaces to generate cell sheets for cardiac repair [70,71]. Intramyocardial injection of PNIPAAm has been shown to reduce collagen deposition, increase neovascularization, limit LV enlargement, and improve cardiac function in a rat MI model [71]. In this study, copolymers synthesized from NIPAAm, 2-Hydroxyethyl methacrylate, and methacrylate-polylactide by free radical polymerization were intramyocardially injected into rat heart after MI [71]. As a result, LV dilation was reduced, and cardiac function was improved in the rat model. Additionally, a biohybrid PNIPAAm-gelatin-based hydrogel improved the survival and maturation of encapsulated cardiac cells in vitro as shown by their superior structural organization and cell-cell coupling [75]. Lastly, hiPSC-CMs and hiPSC-ECs were also seeded successfully in randomly oriented or aligned polycaprolactone scaffolds [72]. The latter significantly improved hiPSC-CM maturation, as well as increased the sarcomeric length and gene expression of myosin heavy chain adult isoform compared with randomly oriented scaffolds.

### 3.4. 3D Printing in Cardiac Tissue Engineering Using PSC Derived Cardiovascular Cells

In recent years, the rapid development of 3D printing technology enabled the construction of hydrogels and myocardial patches into highly precise and repeatable nanoscale 3D structures using multiple cell types. Biomaterials that are used for the 3D printing of myocardial tissue include alginate [93,94], fibrin [94], collagen [95,96], gelatin [97,98,99], hyaluronic acid [98], hydroxypropyl chitin [100], thixotropic magnesium phosphate [101], gellan gum [99], and decellularized ECM scaffolds [102,103,104]. Maiullari et al. manufactured a vascularized heart tissue using human umbilical vein endothelial cell (HUVEC) and miPSC-CMs using alginate and PEG/fibrin hydrogel extruded through a microfluidic printing head [94]. This study successfully generated a functional patch made of miPSC-CMs that aligned along the fiber printing direction. Furthermore, it was also observed that a pre-formed vasculature in 3D heart tissue has the potential to anastomose with the host’s vessels rapidly to supply blood to the implanted sample [94].

Lee et al. described a 3D printing technique to build complex collagen scaffolds for engineering vessels, contractile cardiac ventricle models, trileaflet heart valves, and even neonatal-scale human hearts [95]. Collagen gelation was controlled by modulating the pH and the printing resolution (up to 10 μm). With this technique, cells were successfully embedded in the collagen scaffolds. This is the first study to demonstrate successful 3D printing of a neonatal-sized heart with validated functions.

Fully xeno-free and personalized biomaterials as bioinks for 3D printing have also been reported. Noor et al. developed thick and perfusable cardiac patches using a patient’s omentum-derived hydrogel and self hiPSC-CMs and hiPSC-ECS using 3D bioprinting [105]. Vascularized patches were printed according to the anatomy of the human heart. Strikingly, a small human heart (height: 20 mm; diameter: 14 mm) was created by combining heart CT imaging and mathematical modeling, hiPSC-CMs, and hiPSC-ECs via 3D printing. This study highlights the feasibility and potential of engineering cardiac tissues with natural human anatomical structure and patient-specific biochemical microenvironment.

Considering that most of the biomaterials used so far for bioprinting cannot represent the complexity of the ECM in human tissues or organs, a bioink made of decellularized ECM has been developed [102]. This tissue-specific bioink may provide crucial cues for improving cell engraftment, survival, and function. Bioinks composed of decellularized human heart tissue with either gelatin methacryloyl (GelMA) or GelMA-methacrylated hyaluronic acid (MeHA) hydrogels were developed [104]. It was observed that all bioinks were compatible with hiPSC-CMs and human cardiac fibroblasts. Moreover, printed cardiac constructs were associated with striation formation and connexin-43 expression. 

Although 3D bioprinting has demonstrated the feasibility to print human cardiac tissue and is considered a promising approach for engineering whole hearts, there are still challenges that need to be addressed. These challenges include the development of an ideal bioink, as well as looking for a sustainable source of cardiovascular cells. A natural human decellularized ECM has obvious advantages in constructing a microenvironment for myocardial regeneration similar to natural myocardial tissues in terms of molecular composition, morphological structure, mechanical properties, and electrical conductivity. However, immunogenicity and limited donor sources limit the wide application of acellular heart tissues. Although the well-established hiPSC reprogramming and differentiation techniques can provide enough cardiovascular cells for 3D printing, patient- or disease-specific hiPSC-derived cardiovascular cells may not be suitable as an autologous cell source used in 3D printing. In this case, universal hiPSC-derived cardiovascular cells may be an option. 

## 4. Challenges and Future Directions of Cardiac Tissue Engineering

Although substantial progress has been made in cardiac bioengineering, there are still some challenges that hinder the progress of this promising field:

a. Natural Biomaterials vs. Synthetic Biomaterials

Ideally, cardiac tissue engineering combines biodegradable biomaterials with cardiomyocytes with functional properties such as electronic and mechanical coupling, calcium handling, and force generation. Thus, biodegradable biomaterials, natural or synthetic, with minimal immunogenicity are preferred. Therefore, a xeno-free and autologous cardiac tissue patch will be required as a clinical product to achieve maximal therapeutic benefit.

b. Choice of Cells

Although the current hiPSC techniques can provide many cardiovascular cells for cardiac patch manufacturing, using patients’ autologous hiPSC-derived cells may compromise therapeutic efficacy as most patients with ischemic heart disease have diabetes, hypertension, and hypercholesterolemia. These pathophysiological conditions will affect the quality of hiPSC-derived cells through their epigenetic memories [106]. Derivatives of universal hESCs or hiPSCs are therefore a good option for allogeneic transplantation.

c. Progenitor Cells vs. Terminally Differentiated Cardiovascular Cells

Early cardiovascular progenitors exhibit high plasticity that allow them to differentiate into both cardiac and vascular cells. These cells may be more viable than terminally differentiated cells, especially mature CMs [107], thus enhancing cell engraftment efficacy. Currently, clinical studies of hESCs- SSEA-1^+^ cells have been reported. Cells were seeded into a fibrin scaffold and applied onto the infarct area as an adjunct of coronary artery bypass graft in patients suffering from severe HF [89,90]. Except for one patient who died early post-operation from treatment-unrelated comorbidities, the rest had uneventful recoveries and symptomatically improved, with an increased systolic motion observed in the cell-treated segments. No patient experienced arrhythmias, and no tumor was detected during follow-up.

d. Alignment vs. Non-Alignment

Current 3D printing techniques can provide high resolution in printing and can accurately control cell alignment. However, cardiac muscle fibers are oriented spirally around the circumference of the heart. This anatomic arrangement is due to the complex twisting during heart development and is responsible for contraction [108]. Thus, unsynchronized contraction may deteriorate the LV pump function if a well-aligned cardiac patch is applied to an infarcted myocardium with a different orientation. It is therefore imperative to consider cell alignment in the construction of cardiac patches.

## Figures and Tables

**Table 1:** Advantage and disadvantages of four types of cells used for cellular cardiomyoplasty.

Cell Types	Advantages	Disadvantages
Skeletal myoblasts	Easy to obtain Easy to expand to get large number of cells in vitro Low ethical concerns No risk of tumorigenicity	Risk of inducing ventricular arrhythmias Failure to transdifferentiate into functional cardiomyocytes
Mesenchymal stem cells	Easy to obtain Can be selected by defined cell surface marker Low ethical concerns	Limited cell quantity Limited differentiation potential Cells can only differentiate into cardiomyocyte-like cells
Embryonic stem cells	Pluripotent stemness Well characterized cell lines Theoretically, they can be differentiated into all somatic cells found in the human body	Genetically unstable Risk of tumorigenicity Allogenic transplantation induces immune rejection Ethical issues
Induced pluripotent stem cells	Pluripotent stemness Autologous transplantation avoids immune rejection No ethical concern Theoretically, they can differentiate into all somatic cells found in the human body Disease-specific cell lines for disease modeling	Low induction efficiency Genetically unstable Risk of tumorigenicity Disease-specific cell lines are not suitable for autologous transplantation

## References

[1] Dai H., Zhang Q., Much A.A., Maor E., Segev A., Beinart R., Adawi S., Lu Y., Bragazzi N.L., Wu J. (2021). Global, regional, and national prevalence, incidence, mortality, and risk factors for atrial fibrillation, 1990–2017: Results from the Global Burden of Disease Study 2017. Eur. Heart J. Qual. Care Clin. Outcomes.

[2] Chiong M., Wang Z.V., Pedrozo Z., Cao D.J., Troncoso R., Ibacache M., Criollo A., Nemchenko A., Hill J.A., Lavandero S. (2011). Cardiomyocyte death: Mechanisms and translational implications. Cell Death Dis.

[3] Bergmann O., Zdunek S., Felker A., Salehpour M., Alkass K., Bernard S., Sjostrom S.L., Szewczykowska M., Jackowska T., Dos Remedios C. (2015). Dynamics of Cell Generation and Turnover in the Human Heart. Cell.

[4] Gerber Y., Weston S.A., Enriquez-Sarano M., Berardi C., Chamberlain A.M., Manemann S.M., Jiang R., Dunlay S.M., Roger V.L. (2016). Mortality Associated With Heart Failure After Myocardial Infarction: A Contemporary Community Perspective. Circ. Heart Fail..

[5] Muller P., Lemcke H., David R. (2018). Stem Cell Therapy in Heart Diseases—Cell Types, Mechanisms and Improvement Strategies. Cell Physiol. Biochem..

[6] Katarzyna R. (2017). Adult Stem Cell Therapy for Cardiac Repair in Patients After Acute Myocardial Infarction Leading to Ischemic Heart Failure: An Overview of Evidence from the Recent Clinical Trials. Curr. Cardiol. Rev..

[7] Mirotsou M., Jayawardena T.M., Schmeckpeper J., Gnecchi M., Dzau V.J. (2011). Paracrine mechanisms of stem cell reparative and regenerative actions in the heart. J. Mol. Cell Cardiol..

[8] Kwon S.G., Kwon Y.W., Lee T.W., Park G.T., Kim J.H. (2018). Recent advances in stem cell therapeutics and tissue engineering strategies. Biomater. Res..

[9] Berry J.L., Zhu W., Tang Y.L., Krishnamurthy P., Ge Y., Cooke J.P., Chen Y., Garry D.J., Yang H.-T., Rajasekaran N.S. (2019). Convergences of Life Sciences and Engineering in Understanding and Treating Heart Failure. Circ. Res..

[10] Nguyen A.H., Marsh P., Schmiess-Heine L., Burke P.J., Lee A., Lee J., Cao H. (2019). Cardiac tissue engineering: State-of-the-art methods and outlook. J. Biol. Eng..

[11] Rodrigues I.C.P., Kaasi A., Maciel Filho R., Jardini A.L., Gabriel L.P. (2018). Cardiac tissue engineering: Current state-of-the-art materials, cells and tissue formation. Einstein (Sao Paulo).

[12] Zakrzewski W., Dobrzynski M., Szymonowicz M., Rybak Z. (2019). Stem cells: Past, present, and future. Stem Cell Res. Ther..

[13] Ye L., Haider H., Tan R., Toh W., Law P.K., Tan W., Su L., Zhang W., Ge R., Zhang Y. (2007). Transplantation of nanoparticle transfected skeletal myoblasts overexpressing vascular endothelial growth factor-165 for cardiac repair. Circulation.

[14] Ye L., Haider H., Tan R., Su L., Law P.K., Zhang W., Sim E.K. (2008). Angiomyogenesis using liposome based vascular endothelial growth factor-165 transfection with skeletal myoblast for cardiac repair. Biomaterials.

[15] Ye L., Haider H., Jiang S., Tan R.S., Ge R., Law P.K., Sim E.K. (2007). Improved angiogenic response in pig heart following ischaemic injury using human skeletal myoblast simultaneously expressing VEGF165 and angiopoietin-1. Eur. J. Heart Fail..

[16] Sid-Otmane C., Perrault L.P., Ly H.Q. (2020). Mesenchymal stem cell mediates cardiac repair through autocrine, paracrine and endocrine axes. J. Transl. Med..

[17] Ahmed L.A., Al-Massri K.F. (2021). Directions for Enhancement of the Therapeutic Efficacy of Mesenchymal Stem Cells in Different Neurodegenerative and Cardiovascular Diseases: Current Status and Future Perspectives. Curr. Stem Cell Res. Ther..

[18] Ye L., Zhang P., Duval S., Su L., Xiong Q., Zhang J. (2013). Thymosin beta4 increases the potency of transplanted mesenchymal stem cells for myocardial repair. Circulation.

[19] Xiong Q., Ye L., Zhang P., Lepley M., Swingen C., Zhang L., Kaufman D.S., Zhang J. (2012). Bioenergetic and functional consequences of cellular therapy: Activation of endogenous cardiovascular progenitor cells. Circ. Res..

[20] Yap L., Wang J.W., Moreno-Moral A., Chong L.Y., Sun Y., Harmston N., Wang X., Chong S.Y., Vanezis K., Ohman M.K. (2020). In Vivo Generation of Post-infarct Human Cardiac Muscle by Laminin-Promoted Cardiovascular Progenitors. Cell Rep..

[21] Ye L., Chang Y.H., Xiong Q., Zhang P., Zhang L., Somasundaram P., Lepley M., Swingen C., Su L., Wendel J.S. (2014). Cardiac repair in a porcine model of acute myocardial infarction with human induced pluripotent stem cell-derived cardiovascular cells. Cell Stem Cell.

[22] Tao Z., Loo S., Su L., Tan S., Tee G., Gan S.U., Zhang J., Chen X., Ye L. (2021). Angiopoietin-1 enhanced myocyte mitosis, engraftment, and the reparability of hiPSC-CMs for treatment of myocardial infarction. Cardiovasc. Res..

[23] Tan S.H., Loo S.J., Gao Y., Tao Z.H., Su L.P., Wang C.X., Zhang S.L., Mu Y.H., Cui Y.H., Abdurrachim D. (2021). Thymosin beta4 increases cardiac cell proliferation, cell engraftment, and the reparative potency of human induced-pluripotent stem cell-derived cardiomyocytes in a porcine model of acute myocardial infarction. Theranostics.

[24] Weinberger L., Ayyash M., Novershtern N., Hanna J.H. (2016). Dynamic stem cell states: Naive to primed pluripotency in rodents and humans. Nat. Rev. Mol. Cell Biol..

[25] Nichols J., Smith A. (2009). Naive and primed pluripotent states. Cell Stem Cell.

[26] Downing T.L., Soto J., Morez C., Houssin T., Fritz A., Yuan F., Chu J., Patel S., Schaffer D.V., Li S. (2013). Biophysical regulation of epigenetic state and cell reprogramming. Nat. Mater..

[27] Tan Y., Tajik A., Chen J., Jia Q., Chowdhury F., Wang L., Chen J., Zhang S., Hong Y., Yi H. (2014). Matrix softness regulates plasticity of tumour-repopulating cells via H3K9 demethylation and Sox2 expression. Nat. Commun..

[28] Chen J., Liu H., Liu J., Qi J., Wei B., Yang J., Liang H., Chen Y., Chen J., Wu Y. (2013). H3K9 methylation is a barrier during somatic cell reprogramming into iPSCs. Nat. Genet..

[29] Jaenisch R., Young R. (2008). Stem cells, the molecular circuitry of pluripotency and nuclear reprogramming. Cell.

[30] Kwart D., Paquet D., Teo S., Tessier-Lavigne M. (2017). Precise and efficient scarless genome editing in stem cells using CORRECT. Nat. Protoc..

[31] Hockemeyer D., Jaenisch R. (2016). Induced Pluripotent Stem Cells Meet Genome Editing. Cell Stem Cell.

[32] Weltner J., Balboa D., Katayama S., Bespalov M., Krjutskov K., Jouhilahti E.M., Trokovic R., Kere J., Otonkoski T. (2018). Human pluripotent reprogramming with CRISPR activators. Nat. Commun..

[33] Howden S.E., Thomson J.A., Little M.H. (2018). Simultaneous reprogramming and gene editing of human fibroblasts. Nat. Protoc..

[34] Melo U.S., de Souza Leite F., Costa S., Rosenberg C., Zatz M. (2018). A fast method to reprogram and CRISPR/Cas9 gene editing from erythroblasts. Stem Cell Res..

[35] Poh Y.C., Chen J., Hong Y., Yi H., Zhang S., Chen J., Wu D.C., Wang L., Jia Q., Singh R. (2014). Generation of organized germ layers from a single mouse embryonic stem cell. Nat. Commun..

[36] Zhang S., Dutton J.R., Su L., Zhang J., Ye L. (2014). The influence of a spatiotemporal 3D environment on endothelial cell differentiation of human induced pluripotent stem cells. Biomaterials.

[37] Su L., Kong X., Lim S., Loo S., Tan S., Poh K., Dutton J., Stewart C., Cook S., Su X. (2018). The prostaglandin H2 analog U-46619 improves the differentiation efficiency of human induced pluripotent stem cells into endothelial cells by activating both p38MAPK and ERK1/2 signaling pathways. Stem Cell Res. Ther..

[38] Zanotelli M.R., Ardalani H., Zhang J., Hou Z., Nguyen E.H., Swanson S., Nguyen B.K., Bolin J., Elwell A., Bischel L.L. (2016). Stable engineered vascular networks from human induced pluripotent stem cell-derived endothelial cells cultured in synthetic hydrogels. Acta Biomater..

[39] Ting S., Chen A., Reuveny S., Oh S. (2014). An intermittent rocking platform for integrated expansion and differentiation of human pluripotent stem cells to cardiomyocytes in suspended microcarrier cultures. Stem Cell Res..

[40] Prabhakaran M.P., Mobarakeh L.G., Kai D., Karbalaie K., Nasr-Esfahani M.H., Ramakrishna S. (2014). Differentiation of embryonic stem cells to cardiomyocytes on electrospun nanofibrous substrates. J. Biomed. Mater. Res. B Appl. Biomater..

[41] Parveen S., Singh S.P., Panicker M.M., Gupta P.K. (2019). Amniotic membrane as novel scaffold for human iPSC-derived cardiomyogenesis. In Vitro Cell Dev. Biol. Anim..

[42] Riegler J., Tiburcy M., Ebert A., Tzatzalos E., Raaz U., Abilez O.J., Shen Q., Kooreman N.G., Neofytou E., Chen V.C. (2015). Human Engineered Heart Muscles Engraft and Survive Long Term in a Rodent Myocardial Infarction Model. Circ. Res..

[43] Tiburcy M., Hudson J.E., Balfanz P., Schlick S., Meyer T., Chang Liao M.L., Levent E., Raad F., Zeidler S., Wingender E. (2017). Defined Engineered Human Myocardium With Advanced Maturation for Applications in Heart Failure Modeling and Repair. Circulation.

[44] Bakunts K., Gillum N., Karabekian Z., Sarvazyan N. (2008). Formation of cardiac fibers in Matrigel matrix. Biotechniques.

[45] Xu X., Jha A.K., Harrington D.A., Farach-Carson M.C., Jia X. (2012). Hyaluronic Acid-Based Hydrogels: From a Natural Polysaccharide to Complex Networks. Soft Matter..

[46] Borzacchiello A., Russo L., Malle B.M., Schwach-Abdellaoui K., Ambrosio L. (2015). Hyaluronic Acid Based Hydrogels for Regenerative Medicine Applications. Biomed. Res. Int..

[47] Gao L., Kupfer M.E., Jung J.P., Yang L., Zhang P., Da Sie Y., Tran Q., Ajeti V., Freeman B.T., Fast V.G. (2017). Myocardial Tissue Engineering With Cells Derived From Human-Induced Pluripotent Stem Cells and a Native-Like, High-Resolution, 3-Dimensionally Printed Scaffold. Circ. Res..

[48] McCain M.L., Agarwal A., Nesmith H.W., Nesmith A.P., Parker K.K. (2014). Micromolded gelatin hydrogels for extended culture of engineered cardiac tissues. Biomaterials.

[49] Zhang F., Zhang N., Meng H.X., Liu H.X., Lu Y.Q., Liu C.M., Zhang Z.M., Qu K.Y., Huang N.P. (2019). Easy Applied Gelatin-Based Hydrogel System for Long-Term Functional Cardiomyocyte Culture and Myocardium Formation. ACS Biomater. Sci. Eng..

[50] Ahmadi F., Oveisi Z., Samani S.M., Amoozgar Z. (2015). Chitosan based hydrogels: Characteristics and pharmaceutical applications. Res. Pharm. Sci..

[51] Shariatinia Z., Jalali A.M. (2018). Chitosan-based hydrogels: Preparation, properties and applications. Int. J. Biol. Macromol..

[52] Neves M.I., Moroni L., Barrias C.C. (2020). Modulating Alginate Hydrogels for Improved Biological Performance as Cellular 3D Microenvironments. Front. Bioeng. Biotechnol..

[53] Santana B.P., Nedel F., Piva E., de Carvalho R.V., Demarco F.F., Carreno N.L. (2013). Preparation, modification, and characterization of alginate hydrogel with nano-/microfibers: A new perspective for tissue engineering. Biomed. Res. Int..

[54] Liberski A., Latif N., Raynaud C., Bollensdorff C., Yacoub M. (2016). Alginate for cardiac regeneration: From seaweed to clinical trials. Glob. Cardiol. Sci. Pract..

[55] Kaiser N.J., Kant R.J., Minor A.J., Coulombe K.L.K. (2019). Optimizing Blended Collagen-Fibrin Hydrogels for Cardiac Tissue Engineering with Human iPSC-derived Cardiomyocytes. ACS Biomater. Sci. Eng..

[56] Edalat S.G., Jang Y., Kim J., Park Y. (2019). Collagen Type I Containing Hybrid Hydrogel Enhances Cardiomyocyte Maturation in a 3D Cardiac Model. Polymers.

[57] Qin X., Riegler J., Tiburcy M., Zhao X., Chour T., Ndoye B., Nguyen M., Adams J., Ameen M., Denney T.S. (2016). Magnetic Resonance Imaging of Cardiac Strain Pattern Following Transplantation of Human Tissue Engineered Heart Muscles. Circ. Cardiovasc. Imaging.

[58] Tulloch N.L., Muskheli V., Razumova M.V., Korte F.S., Regnier M., Hauch K.D., Pabon L., Reinecke H., Murry C.E. (2011). Growth of engineered human myocardium with mechanical loading and vascular coculture. Circ. Res..

[59] Nakane T., Masumoto H., Tinney J.P., Yuan F., Kowalski W.J., Ye F., LeBlanc A.J., Sakata R., Yamashita J.K., Keller B.B. (2017). Impact of Cell Composition and Geometry on Human Induced Pluripotent Stem Cells-Derived Engineered Cardiac Tissue. Sci. Rep..

[60] Riemenschneider S.B., Mattia D.J., Wendel J.S., Schaefer J.A., Ye L., Guzman P.A., Tranquillo R.T. (2016). Inosculation and perfusion of pre-vascularized tissue patches containing aligned human microvessels after myocardial infarction. Biomaterials.

[61] Wendel J.S., Ye L., Tao R., Zhang J., Zhang J., Kamp T.J., Tranquillo R.T. (2015). Functional Effects of a Tissue-Engineered Cardiac Patch From Human Induced Pluripotent Stem Cell-Derived Cardiomyocytes in a Rat Infarct Model. Stem Cells Transl. Med..

[62] Querdel E., Reinsch M., Castro L., Kose D., Bahr A., Reich S., Geertz B., Ulmer B., Schulze M., Lemoine M.D. (2021). Human Engineered Heart Tissue Patches Remuscularize the Injured Heart in a Dose-Dependent Manner. Circulation.

[63] Pecha S., Yorgan K., Rohl M., Geertz B., Hansen A., Weinberger F., Sehner S., Ehmke H., Reichenspurner H., Eschenhagen T. (2019). Human iPS cell-derived engineered heart tissue does not affect ventricular arrhythmias in a guinea pig cryo-injury model. Sci. Rep..

[64] Contessotto P., Orbanic D., Da Costa M., Jin C., Owens P., Chantepie S., Chinello C., Newell J., Magni F., Papy-Garcia D. (2021). Elastin-like recombinamers-based hydrogel modulates post-ischemic remodeling in a non-transmural myocardial infarction in sheep. Sci. Transl. Med..

[65] Esser T.U., Trossmann V.T., Lentz S., Engel F.B., Scheibel T. (2021). Designing of spider silk proteins for human induced pluripotent stem cell-based cardiac tissue engineering. Mater. Today Biol..

[66] Dobner S., Bezuidenhout D., Govender P., Zilla P., Davies N. (2009). A synthetic non-degradable polyethylene glycol hydrogel retards adverse post-infarct left ventricular remodeling. J. Card. Fail..

[67] Chow A., Stuckey D.J., Kidher E., Rocco M., Jabbour R.J., Mansfield C.A., Darzi A., Harding S.E., Stevens M.M., Athanasiou T. (2017). Human Induced Pluripotent Stem Cell-Derived Cardiomyocyte Encapsulating Bioactive Hydrogels Improve Rat Heart Function Post Myocardial Infarction. Stem Cell Rep..

[68] Chen Y., Wang J., Shen B., Chan C.W., Wang C., Zhao Y., Chan H.N., Tian Q., Chen Y., Yao C. (2015). Engineering a freestanding biomimetic cardiac patch using biodegradable poly(lactic-co-glycolic acid) (PLGA) and human embryonic stem cell-derived ventricular cardiomyocytes (hESC-VCMs). Macromol. Biosci..

[69] Song S.Y., Kim H., Yoo J., Kwon S.P., Park B.W., Kim J.J., Ban K., Char K., Park H.J., Kim B.S. (2020). Prevascularized, multiple-layered cell sheets of direct cardiac reprogrammed cells for cardiac repair. Biomater. Sci..

[70] Daliri K., Pfannkuche K., Garipcan B. (2021). Effects of physicochemical properties of polyacrylamide (PAA) and (polydimethylsiloxane) PDMS on cardiac cell behavior. Soft Matter.

[71] Ren S., Jiang X., Li Z., Wen Y., Chen D., Li X., Zhang X., Zhuo R., Chu H. (2012). Physical properties of poly(N-isopropylacrylamide) hydrogel promote its effects on cardiac protection after myocardial infarction. J. Int. Med. Res..

[72] Wanjare M., Hou L., Nakayama K.H., Kim J.J., Mezak N.P., Abilez O.J., Tzatzalos E., Wu J.C., Huang N.F. (2017). Anisotropic microfibrous scaffolds enhance the organization and function of cardiomyocytes derived from induced pluripotent stem cells. Biomater. Sci..

[73] Chiono V., Mozetic P., Boffito M., Sartori S., Gioffredi E., Silvestri A., Rainer A., Giannitelli S.M., Trombetta M., Nurzynska D. (2014). Polyurethane-based scaffolds for myocardial tissue engineering. Interface Focus.

[74] Vasile C., Pamfil D., Stoleru E., Baican M. (2020). New Developments in Medical Applications of Hybrid Hydrogels Containing Natural Polymers. Molecules.

[75] Navaei A., Truong D., Heffernan J., Cutts J., Brafman D., Sirianni R.W., Vernon B., Nikkhah M. (2016). PNIPAAm-based biohybrid injectable hydrogel for cardiac tissue engineering. Acta Biomater..

[76] Kadner K., Dobner S., Franz T., Bezuidenhout D., Sirry M.S., Zilla P., Davies N.H. (2012). The beneficial effects of deferred delivery on the efficiency of hydrogel therapy post myocardial infarction. Biomaterials.

[77] Yoshizumi T., Zhu Y., Jiang H., D’Amore A., Sakaguchi H., Tchao J., Tobita K., Wagner W.R. (2016). Timing effect of intramyocardial hydrogel injection for positively impacting left ventricular remodeling after myocardial infarction. Biomaterials.

[78] Ye L., Basu J., Zhang J. (2015). Fabrication of a myocardial patch with cells differentiated from human-induced pluripotent stem cells. Methods Mol. Biol..

[79] Weinberger F., Breckwoldt K., Pecha S., Kelly A., Geertz B., Starbatty J., Yorgan T., Cheng K.H., Lessmann K., Stolen T. (2016). Cardiac repair in guinea pigs with human engineered heart tissue from induced pluripotent stem cells. Sci. Transl. Med..

[80] Case Records of the Massachusetts General Hospital (1997). Weekly clinicopathological exercises. Case 29-1997. A 54-year-old diabetic woman with pain and swelling of the leg. N. Engl. J. Med..

[81] Gao L., Gregorich Z.R., Zhu W., Mattapally S., Oduk Y., Lou X., Kannappan R., Borovjagin A.V., Walcott G.P., Pollard A.E. (2018). Large Cardiac Muscle Patches Engineered From Human Induced-Pluripotent Stem Cell-Derived Cardiac Cells Improve Recovery From Myocardial Infarction in Swine. Circulation.

[82] Menasche P., Vanneaux V., Fabreguettes J.R., Bel A., Tosca L., Garcia S., Bellamy V., Farouz Y., Pouly J., Damour O. (2015). Towards a clinical use of human embryonic stem cell-derived cardiac progenitors: A translational experience. Eur. Heart J..

[83] Fan C., Tang Y., Zhao M., Lou X., Pretorius D., Menasche P., Zhu W., Zhang J. (2020). CHIR99021 and fibroblast growth factor 1 enhance the regenerative potency of human cardiac muscle patch after myocardial infarction in mice. J. Mol. Cell Cardiol..

[84] Kawamura M., Miyagawa S., Miki K., Saito A., Fukushima S., Higuchi T., Kawamura T., Kuratani T., Daimon T., Shimizu T. (2012). Feasibility, safety, and therapeutic efficacy of human induced pluripotent stem cell-derived cardiomyocyte sheets in a porcine ischemic cardiomyopathy model. Circulation.

[85] Kawamura M., Miyagawa S., Fukushima S., Saito A., Miki K., Ito E., Sougawa N., Kawamura T., Daimon T., Shimizu T. (2013). Enhanced survival of transplanted human induced pluripotent stem cell-derived cardiomyocytes by the combination of cell sheets with the pedicled omental flap technique in a porcine heart. Circulation.

[86] Kawamura M., Miyagawa S., Fukushima S., Saito A., Miki K., Funakoshi S., Yoshida Y., Yamanaka S., Shimizu T., Okano T. (2017). Enhanced Therapeutic Effects of Human iPS Cell Derived-Cardiomyocyte by Combined Cell-Sheets with Omental Flap Technique in Porcine Ischemic Cardiomyopathy Model. Sci. Rep..

[87] Xiong Q., Ye L., Zhang P., Lepley M., Tian J., Li J., Zhang L., Swingen C., Vaughan J.T., Kaufman D.S. (2013). Functional consequences of human induced pluripotent stem cell therapy: Myocardial ATP turnover rate in the in vivo swine heart with postinfarction remodeling. Circulation.

[88] Ishigami M., Masumoto H., Ikuno T., Aoki T., Kawatou M., Minakata K., Ikeda T., Sakata R., Yamashita J.K., Minatoya K. (2018). Human iPS cell-derived cardiac tissue sheets for functional restoration of infarcted porcine hearts. PLoS ONE.

[89] Menasche P., Vanneaux V., Hagege A., Bel A., Cholley B., Cacciapuoti I., Parouchev A., Benhamouda N., Tachdjian G., Tosca L. (2015). Human embryonic stem cell-derived cardiac progenitors for severe heart failure treatment: First clinical case report. Eur. Heart J..

[90] Menasche P., Vanneaux V., Hagege A., Bel A., Cholley B., Parouchev A., Cacciapuoti I., Al-Daccak R., Benhamouda N., Blons H. (2018). Transplantation of Human Embryonic Stem Cell-Derived Cardiovascular Progenitors for Severe Ischemic Left Ventricular Dysfunction. J. Am. Coll. Cardiol..

[91] Ban K., Park H.J., Kim S., Andukuri A., Cho K.W., Hwang J.W., Cha H.J., Kim S.Y., Kim W.S., Jun H.W. (2014). Cell therapy with embryonic stem cell-derived cardiomyocytes encapsulated in injectable nanomatrix gel enhances cell engraftment and promotes cardiac repair. ACS Nano.

[92] Xu X., Liu Y., Fu W., Yao M., Ding Z., Xuan J., Li D., Wang S., Xia Y., Cao M. (2020). Poly(N-isopropylacrylamide)-Based Thermoresponsive Composite Hydrogels for Biomedical Applications. Polymers.

[93] Gaetani R., Doevendans P.A., Metz C.H., Alblas J., Messina E., Giacomello A., Sluijter J.P. (2012). Cardiac tissue engineering using tissue printing technology and human cardiac progenitor cells. Biomaterials.

[94] Maiullari F., Costantini M., Milan M., Pace V., Chirivi M., Maiullari S., Rainer A., Baci D., Marei H.E., Seliktar D. (2018). A multi-cellular 3D bioprinting approach for vascularized heart tissue engineering based on HUVECs and iPSC-derived cardiomyocytes. Sci. Rep..

[95] Lee A., Hudson A.R., Shiwarski D.J., Tashman J.W., Hinton T.J., Yerneni S., Bliley J.M., Campbell P.G., Feinberg A.W. (2019). 3D bioprinting of collagen to rebuild components of the human heart. Science.

[96] Tytgat L., Dobos A., Markovic M., Van Damme L., Van Hoorick J., Bray F., Thienpont H., Ottevaere H., Dubruel P., Ovsianikov A. (2020). High-Resolution 3D Bioprinting of Photo-Cross-linkable Recombinant Collagen to Serve Tissue Engineering Applications. Biomacromolecules.

[97] AnilKumar S., Allen S.C., Tasnim N., Akter T., Park S., Kumar A., Chattopadhyay M., Ito Y., Suggs L.J., Joddar B. (2019). The applicability of furfuryl-gelatin as a novel bioink for tissue engineering applications. J. Biomed. Mater. Res. B Appl. Biomater..

[98] Gaetani R., Feyen D.A., Verhage V., Slaats R., Messina E., Christman K.L., Giacomello A., Doevendans P.A., Sluijter J.P. (2015). Epicardial application of cardiac progenitor cells in a 3D-printed gelatin/hyaluronic acid patch preserves cardiac function after myocardial infarction. Biomaterials.

[99] Koivisto J.T., Gering C., Karvinen J., Maria Cherian R., Belay B., Hyttinen J., Aalto-Setala K., Kellomaki M., Parraga J. (2019). Mechanically Biomimetic Gelatin-Gellan Gum Hydrogels for 3D Culture of Beating Human Cardiomyocytes. ACS Appl. Mater. Interfaces.

[100] Li Y., Jiang X., Li L., Chen Z.N., Gao G., Yao R., Sun W. (2018). 3D printing human induced pluripotent stem cells with novel hydroxypropyl chitin bioink: Scalable expansion and uniform aggregation. Biofabrication.

[101] Chen Y., Wang Y., Yang Q., Liao Y., Zhu B., Zhao G., Shen R., Lu X., Qu S. (2018). A novel thixotropic magnesium phosphate-based bioink with excellent printability for application in 3D printing. J. Mater. Chem. B.

[102] Pati F., Jang J., Ha D.H., Won Kim S., Rhie J.W., Shim J.H., Kim D.H., Cho D.W. (2014). Printing three-dimensional tissue analogues with decellularized extracellular matrix bioink. Nat. Commun..

[103] Kc P., Hong Y., Zhang G. (2019). Cardiac tissue-derived extracellular matrix scaffolds for myocardial repair: Advantages and challenges. Regen. Biomater..

[104] Basara G., Ozcebe S.G., Ellis B.W., Zorlutuna P. (2021). Tunable Human Myocardium Derived Decellularized Extracellular Matrix for 3D Bioprinting and Cardiac Tissue Engineering. Gels.

[105] Noor N., Shapira A., Edri R., Gal I., Wertheim L., Dvir T. (2019). 3D Printing of Personalized Thick and Perfusable Cardiac Patches and Hearts. Adv. Sci..

[106] Su L., Kong X., Loo S.J., Gao Y., Kovalik J.P., Su X., Ma J., Ye L. (2021). Diabetic Endothelial Cells Differentiated From Patient iPSCs Show Dysregulated Glycine Homeostasis and Senescence Associated Phenotypes. Front. Cell Dev. Biol..

[107] Tan S.H., Ye L. (2018). Maturation of Pluripotent Stem Cell-Derived Cardiomyocytes: A Critical Step for Drug Development and Cell Therapy. J. Cardiovasc. Transl. Res..

[108] Anderson R.H., Ho S.Y., Redmann K., Sanchez-Quintana D., Lunkenheimer P.P. (2005). The anatomical arrangement of the myocardial cells making up the ventricular mass. Eur. J. Cardiothorac. Surg..

